# Outcomes and Recommendations of an Indian Expert Panel for Improved Practice in Controlled Ovarian Stimulation for Assisted Reproductive Technology

**DOI:** 10.1155/2017/9451235

**Published:** 2017-01-26

**Authors:** Baiju Ahemmed, Vani Sundarapandian, Rohit Gutgutia, Sathya Balasubramanyam, Richa Jagtap, Reeta Biliangady, Priti Gupta, Sachin Jadhav, Ruma Satwik, Pavitra Raj Dewda, Priti Thakor, Sandro C. Esteves

**Affiliations:** ^1^NCARE Group of IVF Centres, Kerala, India; ^2^Jananam Fertility Center, Chennai, India; ^3^Nova IVI Fertility, Kolkata, India; ^4^Cloud 9 Hospitals, Chennai, India; ^5^Nova IVI Fertility, Mumbai, India; ^6^Cloud 9 Fertility, Bengaluru, India; ^7^Fertility and IVF-Jaipur Golden Hospital & Gupta Maternity Home, Delhi, India; ^8^Gupte Hospital, Pune, India; ^9^Centre of IVF and Human Reproduction, Sir Ganga Ram Hospital, New Delhi, India; ^10^Medical Affairs, Merck Biopharma, Mumbai, India; ^11^ANDROFERT, Andrology and Human Reproduction Clinic, Referral Center for Male Reproduction, Campinas, SP, Brazil

## Abstract

*Purpose.* To improve success of in vitro fertilization (IVF), assisted reproductive technology (ART) experts addressed four questions. What is optimum oocytes number leading to highest live birth rate (LBR)? Are cohort size and embryo quality correlated? Does gonadotropin type affect oocyte yield? Should “freeze-all” policy be adopted in cycles with progesterone >1.5 ng/mL on day of human chorionic gonadotropin (hCG) administration?* Methods.* Electronic database search included ten studies on which panel gave opinions for improving current practice in controlled ovarian stimulation for ART.* Results.* Strong association existed between retrieved oocytes number (RON) and LBRs. RON impacted likelihood of ovarian hyperstimulation syndrome (OHSS). Embryo euploidy decreased with age, not with cohort size. Progesterone > 1.5 ng/dL did not impair cycle outcomes in patients with high cohorts and showed disparate results on day of hCG administration.* Conclusions.* Ovarian stimulation should be designed to retrieve 10–15 oocytes/treatment. Accurate dosage, gonadotropin type, should be selected as per prediction markers of ovarian response. Gonadotropin-releasing hormone (GnRH) antagonist based protocols are advised to avoid OHSS. Cumulative pregnancy rate was most relevant pregnancy endpoint in ART. Cycles with serum progesterone ≥1.5 ng/dL on day of hCG administration should not adopt “freeze-all” policy. Further research is needed due to lack of data availability on progesterone threshold or index.

## 1. Introduction

Infertility constitutes a major health problem across all ages in India. As a result, the demand for medically assisted modalities to alleviate infertility is growing [1–3]. Since the birth of first baby through natural in vitro fertilization (IVF) in 1978, more than 5 million births have taken place worldwide through assisted reproductive technology (ART) till date [2, 4]. Given that oocytes are the raw material to be fertilized and generate embryos, which shall be available for transfer or cryopreservation, an optimal number of oocytes must be obtained at the end of ovarian stimulation, below and above which, outcomes may be compromised [5]. Interestingly, in 1980, Edwards reported one clinical pregnancy per 11 oocytes retrieved, and one live birth per 15 oocytes retrieved [6]. Owing to the developments in ART in successive years, higher pregnancy rates are reported nowadays but the efficiency of oocyte utilization has not improved significantly [7]. Due to relatively low efficiency of ART, controlled ovarian hyperstimulation (COH) with gonadotropins has been an integral element of treatment and is used to promote multiple follicular growths [8]. COH is used to obtain an increased number of oocytes but a consensus on optimum number of oocytes to be retrieved for a better live birth rate (LBR) is not yet clear [9]. Moreover, the impact of number (cohort size) of oocytes on the quality of embryo is crucial to know.

The ability of the oocyte to be fertilized is largely determined by the morphological and functional changes that are caused by hormonal events, specifically the fluctuation of gonadotropin hormones. Follicle-stimulating hormone (FSH) and luteinizing hormone (LH) work in a complementary manner to regulate the follicle that lead to a synergistic action of stimulating follicular growth and ovulation [10]. However, there is a lack of precision on the effect of different gonadotropins on oocyte yield in IVF.

Many studies have reported an elevated serum progesterone (P) levels above a threshold level of >1.5 ng/mL on the day of human chorionic gonadotropin (hCG) administration [11, 12]. Conflicting views have emerged on the rise of serum P levels with some researchers reporting that the raised levels might be detrimental and thus the embryos should be frozen (“freeze all”) whereas others suggest that elevated P levels are associated with a good ovarian response [13, 14]. In a prospective, randomized trial conducted by Smitz and coworkers, higher P levels with recombinant FSH (rFSH) compared with highly purified menotropin (HP-hMG) even after adjusting for ovarian response were observed [15]. In another randomized, open-labeled, noninferiority trial, Devroey et al. found almost similar levels of P with both rFSH and HP-hMG [16]. Given the nonuniform sensitivity of P measurement assays, especially in the low range of P, there is a lack of clarity among IVF specialists regarding adopting “freeze-all” policy in all cycles with P elevation on day of hCG administration based on a single cut-off point, namely, 1.5 ng/mL. Notwithstanding, among the techniques to measure the P levels during stimulation, isotope dilution-gas chromatograph/mass spectrometry (ID-GC/MS) is a sensitive measurement technique [17].

Evidence from outside India has been recently published to answer questions regarding the optimum number of retrieved oocytes to increase likelihood of LBR with fewer complications, the effect of cohort size on embryo quality, impact of different gonadotropins on oocyte yield, and P levels on the day of hCG administration on the chances of pregnancy. However, a single source that could guide the practitioners in the field of IVF related-infertility on contemporary topics is not available. Thus, the experts in the field of IVF with the help of an International moderator formed a panel to discuss these issues of high clinical relevance based on current literature and their own experiences. This article presents the outcomes and recommendations of the expert panel on these IVF related clinically relevant issues.

## 2. Materials and Methods

Nine Indian infertility experts having at least 10 years of clinical experience and academic contribution (defined by scholar contributions including peer-reviewed articles, book chapters, conference papers, and/or teaching activities) and one international moderator having over 20 years of experience were invited and accepted the task of forming a panel gathered in April 2015, to discuss important clinical issues to IVF practitioners. Among many important issues, the issues considered most pertinent to practitioners were identified a priori based on a survey among panelists. Four clinical questions have been elaborated a priori based on consensus among participants to be discussed in the expert panel meeting. (i) What is the optimum number of oocytes that is associated with the highest LBR? (ii) Is there a correlation between cohort size and embryo quality? (iii) Does the choice of gonadotropins affect oocyte yield in IVF? (iv) Should “freeze-all” policy be adopted in all cycles with P levels >1.5 ng/mL on the day of hCG administration?

In preparation, an extensive literature search with keywords as “oocytes”, “live birth rate”, “assisted reproductive technology”, “in vitro fertilization”, “intracytoplasmic sperm injection”, “recombinant FSH”, “progesterone levels”, and “gonadotropin” was performed in MEDLINE, Cochrane Library, and ScienceDirect databases from January 2011 to April 2015 to identify relevant articles to respond to the clinical questions. The keywords were provided by the moderator and expert panel and the literature search was conducted by the medical writer. Full articles published in English and in peer-reviewed and indexed journals were selected. The moderator independently screened the studies for inclusion. All the potentially relevant articles that could answer the relevant questions were examined, and these were subsequently screened by other reviewers. Discrepancies, if any, were resolved by discussion among the panel members.

## 3. Results

**Figure 1:**
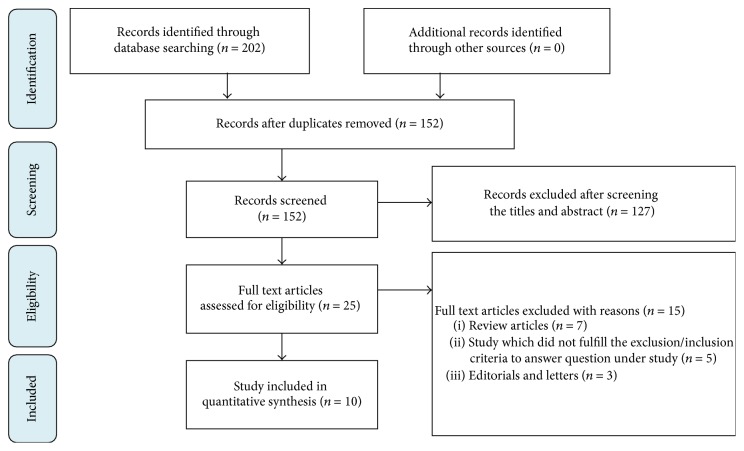
Flowchart for trial identification and selection process using the PRISMA statement for systematic review.

The search retrieved 202 citations, of which 25 were considered for full-text screening and 10 were selected for discussion by the panel (Figure 1). The questions were finalized or decided on the basis of problem/difficulty/confusion faced by the IVF experts while performing IVF/ARTs practically. Excluded articles had poorly defined methodology and/or conflicting or noncommittal results that did not allow answering the proposed clinical questions. In addition, three studies on the influence of P levels on the probability of pregnancy in IVF were excluded due to (i) utilization of P measurement in nmol/L and cut-off levels other than 1.5 ng/mL (or 4.77 nmol/L) [18, 19] and (ii) P determination on a day other than the day of hCG administration [20]. The attributes of the included articles are tabulated in Table 1. Apart from these studies, some studies (cited in the discussion) were also included to support the recommendations of the panel. These articles were referred by individual panelist during the course of discussion and thus used.

### 3.1. What Is the Optimum Number of Oocytes Associated with the Highest LBR?

Sunkara and colleagues in their study demonstrated a firm relationship between the number of oocytes retrieved and LBR. The authors found that LBR increased as the number of oocytes also increased up to 15, stabilized between 15 and 20 oocytes, and steadily declined beyond 20 oocytes across all female age groups [9]. In their study, the median number of oocytes that were collected was 9 [interquartile range (IQR) 6–13]. Overall, the LBR was 21.3% [95% confidence interval (CI): 21.2 to 21.4%]. The authors speculated that a higher number of oocytes could lead to a decreased LBR because of raised serum estradiol levels that have a deleterious effect on embryo implantation.

Subsequently, Ji and colleagues retrospectively evaluated 2,455 patients who had undergone IVF treatments in China. The subjects were categorized into four groups on the basis of the number of oocytes retrieved (0–5, 6–10, 11–15, and >15 oocytes). In this study, the authors analyzed the relationship between oocyte number and LBR, considering not only fresh cycles but also frozen-thawed cycles, thus allowing estimation of cumulative LBR. In fresh transfers, LBR was optimized when the number of oocytes retrieved was 11–15. The odds of LBR increased from 1.823 (95% CI: 1.395 to 2.381) in 6–10 oocyte category to 2.142 (95% CI: 1.609 to 2.851) in 11–15 oocyte category but decreased in ≥16 oocyte category, being 1.918 (95% CI: 1.376 to 2.672). On combining the fresh and frozen cycles, cumulative LBR per initiated cycle increased with the number of oocytes, but the incidence of moderate and severe ovarian hyperstimulation syndrome (OHSS) also increased. The study suggested that the ideal number of oocytes for achieving highest cumulative LBR (CPR) while avoiding risk of OHSS should range between 6 and 15 [21].

Recently, De Geyter and colleagues compared data generated from over 100,000 IVF cycles performed between 1993 and 2012. The Federal Office of Statistics published this data based on the Swiss database on ART. A panel of experts extracted recommendations from these analyses to improve the current practice, prevent complications related to ART, and further recommend modifications in the current Swiss legislation regarding ART. Among all treated patients, the delivery rates per embryo transfer were dependent on the number of oocytes retrieved (*p* < 0.0001), but among younger patients, this association was less pronounced. It was observed that the number of deliveries was significantly lower if <5 oocytes (irrespective of the age groups) were retrieved and did not improve further if >15 oocytes were collected (except >39 years). The quantity of oocytes retrieved determined the likelihood of delivery, risk of multiple births, and incidence of OHSS. As such, the expert panel concluded that an optimum COH for the retrieval of 10 to 15 oocytes per treatment cycle should be designed [22].

### 3.2. Is There a Correlation between Cohort Size and Embryo Quality?

Ata et al. analyzed 7,753 embryos of 990 women (<35 years, good ovarian reserve) undergoing IVF by an array-based comparative genomic hybridization (aCGH). In a linear regression analysis, a total of 5,918 cleavage-stage embryos and 1,218 blastocysts obtained from 726 and 218 women, respectively, were analyzed with aCGH. The authors observed that, for each year increase in female age, euploidy rate decreased by 2.9 percentage points in both day 3 and blastocysts (95% CI: −3.8% to −2.0%, *p* < 0.001), and the odds of achieving at least one euploid embryo were decreased as a function of increased female age in both groups (odds ratio (OR) 0.82, 95% CI 0.70–0.94, *p* = 0.006). Interestingly, the quantity of embryos was not associated with the euploidy rate (*B* = −0.32%, 95% CI: −1.4% to 0.8%). Further, the odds of having a minimum of one euploid embryo significantly increased with every additional embryo available for analysis (OR: 1.55, 95% CI: 1.25 to 1.93, *p* < 0.001). The authors concluded that euploidy significantly decreased with age but not with the cohort size [23].

### 3.3. Do Gonadotropin Isoforms Affect Oocyte Yield in IVF?

In a meta-analysis, Lehert and colleagues reported that hMG resulted in significantly fewer oocytes than recombinant human FSH (r-hFSH) (mean 9.4 ± 6.3 versus 10.9 ± 6.6; mean difference [MD]: −1.54; 95% CI: −2.53 to −0.56; *p* < 0.0001), although a significant heterogeneity was observed among the studies (I^2^ = 63%, *p* = 0.0004). After adjusting for potential confounders, including age, basal FSH, body mass index (BMI), and the number of follicles, the MD was −2.10 (95% CI: −2.83 to −1.36; *p* < 0.001). Further, a higher total dose of hMG was needed during COH compared with r-hFSH (MD: 235.46 IU [95% CI: 16.62 to 454.30; *p* = 0.03]) [24]. The authors estimated ratio of the number of oocytes/1000 IU of gonadotropin dose to be 4.39 and 5.10 for hMG and r-hFSH, respectively, with a mean difference favoring r-FSH of 0.70 oocytes/1000 IU (95% CI: 0.10 to 1.30; *p* = 0.021). The pregnancy rate did not differ significantly between the two gonadotropin regimens for baseline unadjusted and adjusted estimates.

In a subsequent meta-analysis of 40 trials, including 6,443 women, Lehert and colleagues compared the effect of combining recombinant gonadotropins, FSH and LH, with r-hFSH alone on oocyte yield and pregnancy rates. Prospective, parallel, and randomized controlled trials (RCTs) in women aged 18–45 years undergoing IVF, intracytoplasmic sperm injection (ICSI), or both treated with gonadotropin-releasing hormone (GnRH) analogues were included. Overall, no significant difference was observed in the quantity of oocytes retrieved between the r-hFSH + recombinant human LH (r-hLH) and r-hFSH groups (weighted MD −0.03; 95% CI: 0.41 to 0.34). Higher clinical pregnancy rates were achieved with the combination of r-hFSH and r-hLH versus r-hFSH alone in the overall population analyzed (risk ratio [RR]: 1.09; 95% CI: 1.01 to 1.18; intention-to-treat [ITT] population). The RR for the ongoing pregnancy rate (14 studies; RR: 1.14; 95% CI: 1.05 to 1.25) and LBR (8 studies; RR: 1.11; 95% CI: 1.01 to 1.21; ITT population) was significant in favor of the gonadotropin combination. However, a nonsignificant benefit for r-hFSH + r-hLH for ongoing pregnancy rate (OPR) (RR: 1.13; 95% CI: 1.00 to 1.27) and LBR (RR: 1.10; 95% CI: 0.94 to 1.29) was observed in normal responders [25].

Data on poor responders (POR) were available from 14 studies in the aforementioned meta-analysis, and a subgroup analysis of this patient population was carried out. Poor responders were defined according to study authors' criteria, and in 10 of the 14 included studies, the definition of POR was aligned with the reported Bologna criteria (European Society of Human Reproduction and Embryology (ESHRE)) [26]. In the overall population of poor responders, a significant high number of oocytes were collected with r-hFSH + r-hLH versus r-hFSH alone (*n* = 1077; weighted MD: +0.75 oocytes; 95% CI 0.14 to 1.36). A stronger benefit was found in the subgroup of patients who were younger (<36 years of age) poor responders and received GnRH agonist for pituitary desensitization (MD: +1.40 oocytes; 95% CI: 0.35 to 2.46; *p* = 0.01). The authors also noted a significantly higher clinical pregnancy rate with the combination of recombinant gonadotropins versus r-hFSH alone (RR: 1.30; 95% CI: 1.01 to 1.67; ITT population). Data about the OPR was available in 11 studies (1043 patients); among these, a significant benefit was observed overall for r-hFSH + r-hLH (RR: 1.36; 95% CI: 1.04 to 1.79). A nonsignificant benefit for r-hFSH + r-hLH on LBR was observed in the subgroup of poor responders (RR: 1.30; 95% CI 0.95 to 1.78) [25].

### 3.4. Should “Freeze-All” Policy Be Adopted in All Cycles with P Levels >1.5 ng/mL on Day of hCG?

Xu et al. reported the association between serum P levels on the day of hCG administration and OPRs in 11,055 women subjected to their first IVF/ICSI cycles. Their study involved different responders undergoing fresh transfers, including high (≥20 oocytes; *n* = 2023), poor (≤4 oocytes; *n* = 827), or intermediate (5–19 oocytes; *n* = 8,205) according to the quantity of oocytes harvested, as well as 4,021 women subjected to frozen-thawed embryo transfer (FET) cycles. The mean serum levels of P on the day of hCG administration were 1.51 ± 0.51 ng/mL, but they noted a significantly higher serum P levels in the high ovarian response subgroup than in the intermediate and poor ovarian response subgroups (1.89 ± 0.66 ng/mL, 1.47 ± 0.47 ng/mL, and 1.18 ± 0.48 ng/mL, respectively; *p* < 0.001). The women in poor ovarian response group had the lowest P levels compared to other groups (*p* < 0.001). In a multivariate logistic regression analysis, the number of oocytes retrieved, total FSH dose, and serum estradiol values were positively associated with increased P levels on the day of hCG administration. After stratifying patients into eight groups according to serum P levels on the day of hCG administration (<1.00, 1.00–1.25, 1.25–1.50, 1.50–1.75, 1.75–2.00, 2.00–2.25, 2.25–2.5, and >2.5 ng/mL), they observed statistically significant reduced OPR according to the ovarian response category. When serum P was >2.25 ng/mL, there was a statistically significant reduced OPR (27.5% and 36.8%) in high responders (OR: 0.47; 95% CI: 0.26 to 0.85), although the fertilization and cleavage rates were not compromised. For the intermediate and poor responders, the threshold levels to discriminate patients with reduced OPR were 1.75 ng/mL (OR: 0.78; 95% CI: 0.65 to 0.95) and 1.5 ng/mL (OR: 0.36; 95% CI: 0.14 to 0.94), respectively. The percentage of subjects with elevated P levels on the day of hCG was 24.2% overall, and 21.5%, 25.6%, and 17.0% in the categories of high, intermediate, and poor responders, respectively. In contrast to fresh cycles, elevated serum P levels did not significantly influence fertilization, cleavage rates, and OPR in FET cycles [27].

Subsequently, Griesinger and colleagues analyzed ovarian response and OPR based on serum P levels ≤1.5 ng/mL and >1.5 ng/mL on the day of hCG administration by pooling six trials including 1,866 women subjected to IVF with r-hFSH and GnRH antagonist. They observed that women with elevated P (>1.5 ng/mL) had a higher ovarian response and higher quantity of oocytes retrieved as compared to women who had P <1.5 ng/mL (*p* < 0.01). The OR of ongoing pregnancy per embryo transfer for P category (>1.5 ng/mL versus <1.5 ng/mL) on the day of hCG was 0.56 (95% CI: 0.37 to 0.83) after adjusting for trial, age, duration of stimulation, quantity of oocytes harvested, and number of transferred embryos. However, no detrimental effect of P elevation (>1.5 ng/mL) was observed on OPR (39.2% versus 42.3%) in women with high ovarian response (>18 oocytes). The percentage of patients with elevated P levels on the day of hCG was 8.4% overall, and 4.5%, 3.9%, 8.3%, 12.1%, and 19.0% in women with 1–5, 6–9, 10–13, 14–18, and >18 oocytes retrieved, respectively [28]. The aforementioned authors evaluated the impact on OPR if the negative effect of P elevations could have been prevented and estimated that it would have theoretically bring about an increase in the overall pregnancy rate of 1.0 percentage points, that is, from 32.5% to 33.5% in their studied population.

In another study, Requena et al. evaluated the impact of P levels on the day of hCG on IVF outcomes in 2850 women with high ovarian response undergoing IVF in 11 institutions over a 2-year period. High response was defined as women who had ≥20 oocytes harvested or whose estradiol levels were ≥3000 pg/mL. The patients were grouped as per their P levels on the day of hCG as follows: (i) <0.5 ng/mL, (ii) 0.50–0.70 ng/mL, (iii) 0.71–1.00 ng/mL, (iv) 1.01–1.40 ng/mL, (v) 1.41–1.80 ng/mL, and (vi) >1.81 ng/mL. They observed an association between elevated P levels and estradiol levels. Further, no significant differences were noted in the mean P concentration as a function of the type of gonadotropin used for COH: r-hFSH alone (*n* = 728, P 1.06 ng/mL), r-hFSH + r-hLH (*n* = 377, P 1.01 ng/mL), HP-hMG alone (*n* = 370; P 1.10 ng/mL), and r-hFSH + HP- hMG (*n* = 1375; P 1.30 ng/mL). At concentration >1.8 ng/mL (but not at 1.5 ng/mL), the effect of P rise on the probability of pregnancy was minimum (OR: 0.73, 95% CI 0.61 to 0.99) and negligible on the rates of implantation in patients with a high ovarian response. The authors suggested that, in high responders, P levels cannot be used to predict clinical outcomes as depicted by the uninformative value in the area under the curve (AUC) derived from receiver operating characteristics (ROC) analysis [14].

Recently, Venetis and colleagues estimated the impact of P levels on the day of hCG on LBR by quantifying the effect of most important known confounders using a multivariate regression analysis. These confounders included age of the women and the total number of oocytes retrieved, quantity of embryos that are transferred and their developmental stage, presence of a minimum one good-quality embryo transferred, BMI of woman, total dosage of FSH used, and the type of GnRH analogues (agonists versus antagonists) for ovarian stimulation. The authors observed that LBR was similar between cycles with higher and lower P level (<1.5 ng/mL) when a bivariate analysis was performed (OR: 0.78, 95% CI: 0.56 to 1.09). However, in a multivariable analysis controlling for the effect of the confounders, LBR (OR: 0.68, 95% CI 0.48 to 0.97) was significantly decreased in patients with higher P levels on the day of hCG. When the analysis was performed based on quantity of oocytes retrieved, nothing significant could be found in cycles with <6 oocytes and >18 oocytes. However, a negative impact of P levels on LBR was detected that achieved a statistical significance in normal responders (6–18 oocytes, *n* = 1770 cycles) when 0.9 ng/mL, 1.2 ng/mL, and 1.5 ng/mL were used as threshold levels [29]. In this aforementioned study, P elevation (>1.5 ng/mL) on the day of hCG was observed in 243 cycles (7.4%, 95% CI: 6.5%–8.3%). Based on the quantity of oocytes retrieved, the incidence of P elevation (>1.5 ng/mL) was 1.4% (95% CI 0.8% to 2.5%) in patients with <6 oocytes, 6.3% (95% CI 5.3% to 7.6%) in patients with 6–18 oocytes, and 16.5% (95% CI 13.9% to 19.3%) in those with >18 oocytes.

## 4. Discussion

**Figure 2:**
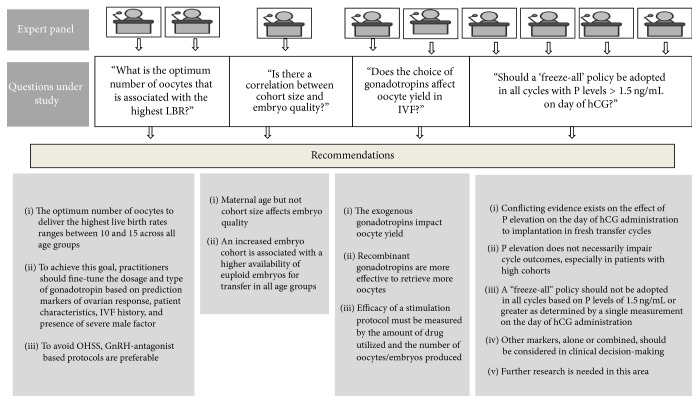
Composition of expert panel, questions under study, and recommendations.

Despite the notable advancement in ART, practitioners still face many challenges in daily clinical practice to properly individualize COH. While the birth of a healthy offspring is the ultimate goal, treatment strategies, including ovarian stimulation, should be planned to avoid serious complications such as multiple deliveries and OHSS. Although multiple drug options and treatment protocols are currently available, the age of patients pursuing ART is continually rising which poses an additional barrier to success. Discontented with these issues, a panel of ART experts from India was formed to formulate a consensus to answer relevant clinical questions related to optimal personalized outcome in COH. This review focused on four interrelated clinical questions, and the results of this panel meeting demonstrated that a consensus was reached among specialists on how to increase effectiveness of ART with regard to COH as discussed below (Figure 2).

The first question focused upon the association of the ideal quantity of oocytes and highest LBR. In 2010, a systematic review and meta-analysis of 14 studies that included >30000 patients found a robust association between the number of oocytes (OR: 1.04, 95% CI 1.02 to 1.07) and pregnancy rate in IVF, whereas female age [OR: 0.95, 95% CI 0.94 to 0.96] was negatively associated with chance of pregnancy [30]. Unfortunately, the authors did not estimate the optimum number of oocytes to be targeted in a given COH attempt, which is crucial as too low number might not result in IVF success and too high number might lead to additional risk factors like OHSS.

The answer to the question was provided by two independent studies involving large data registries in the United Kingdom and Switzerland. Sunkara and colleagues reported that, in different age groups,* namely*, 18–34, 35–37, 38–39, and ≥40 years, the best chance of live birth in an ART is when the number of eggs retrieved is around 15. In cycles involving fresh embryo transfers, LBR declines with an increase in number of eggs beyond 20 [9]. The LBRs were found to decrease not only with female age, as expected, but also with increasing number of retrieved oocytes (>20). The latter could be due to the harmful effect of elevated serum estradiol levels that in turn affect embryo implantation and increase the risk of OHSS [31–34]. The authors thus demonstrated an association between retrieved oocytes and live birth in a fresh IVF cycle across all age groups, suggesting the quantity of oocytes, in addition to age, to be a reasonable surrogate marker in IVF success. They also generated a nomogram which linked the predicted quantity of oocytes to live birth, likely facilitating the planning of COH to both optimize outcomes and prevent complications due to production of excessive oocytes. Along the same lines, De Geyter and colleagues found that outcome of ART strongly depend on the female age and number of collected oocytes [22]. It was observed that the quantity of retrieved oocytes significantly affected the delivery rates per fresh embryo transfer (*p* < 0.0001), but this dependence was less important among younger patients. Interestingly, the number of deliveries was significantly lower in each age category if less than five oocytes were harvested. In all the age categories (except >39 years), the delivery rates did not improve further, if >15 oocytes were collected. Further, a significant rise in the incidence of OHSS was reported if >14 oocytes were collected, regardless of age of the patient. Overall, 17.8% of the embryo transfers among all the initiated treatment cycles were cancelled (irrespective of the age groups), if fewer than 5 but, more frequently, if ≥19 oocytes were harvested. The consensus is also supported by Ji and colleagues, who added by showing that the number of retrieved oocytes is associated with cumulative pregnancy rates, as significantly higher CPR (59% versus 35%) has been obtained with 11–15 oocytes retrieved compared with <6 oocytes [21].

Collectively, these evidences clearly indicate that the quantity of retrieved oocytes is a positive predictor of live birth in IVF. In addition, increased number of oocytes and embryos will be associated with higher cumulative pregnancy and LBR. Our expert panel concluded that the optimum number of oocytes to deliver highest LBR ranges between 10 and 15, and whenever possible, stimulation protocols should be designed to achieve this goal. There is a high variability in the potency of a woman's ovaries in response ovarian stimulation and thus, the recommended strategy to obtain this optimum number of oocytes would be to tailor the dosage and type of gonadotropin according to the prediction markers of ovarian response to stimulation, particularly antimullerian hormone (AMH) and antral follicle count (AFC), in addition to patient clinical characteristics, previous history of COH response, and presence of severe male factor infertility [35–40]. To avoid the occurrence of OHSS, we recommended the use of GnRH antagonists in addition to refining the appropriate dosage of gonadotropin. Notwithstanding, it is important to recognize that individual practices may vary depending on the conditions in which the fertility doctor has to work. Whether vitrification technology is available, single embryo transfer, or the culture strategy (D3 or blastocyst), might influence the stimulation strategy.

Increased numbers of retrieved oocytes resulting from tailored COH are important to optimize LBR, as discussed in the previous section. Provided that the embryo quality is critical to IVF success, the next relevant question that the practitioners would like to know is whether an increased oocyte cohort affects the oocyte quality, and consequently, the embryo quality.

Although conventional embryo morphology, including blastomere number and size as well as cytoplasmic fragmentation, still remains the most used criteria for assessing embryo quality, it is limited as a surrogate marker of embryo viability [41]. Poor embryo morphology may reflect oocytes with compromised development competence, but a “so-called” normal embryo may also carry chromosomal abnormalities [42]. In addition, conventional morphological assessment is both subjective and time-dependent and therefore may lead to erroneous discrimination of embryos with the best implantation ability [41, 43, 44].

Given aneuploidy is the foremost cause of implantation failure, screening for genetic competence of developing embryos remains the gold standard method for assessing embryo quality [45, 46]. Notwithstanding, this does not mean that an euploid embryo will necessarily implant and develop as some other reasons such as inappropriate endometrial receptivity might also cause implantation failure even in normal embryos [47–50].

Among the methods to assess embryo genetic profile, fluorescence in situ hybridization (FISH), aCGH, and more recently, next generation sequencing (NGS) have been utilized [51, 52]. Preimplantation genetic screening (PGS) of the embryos has been found to be a successful technique to analyze aneuploidy, specifically in females with advanced maternal age (>36 years) and those with repetitive implantation failures [53–55].

In a study of 1,255 cleavage-stage human embryos using FISH, aneuploidy increased as a function of female age, from 3.1% in embryos from 20–34 years old patients to 17% in patients 40 years or older. But only chromosomes X, Y, 13, 16, 18, and 21 were analyzed, as FISH allows analysis of a limited number of chromosomes [56]. In contrast, aCGH has been shown to be technologically better and more precise technique for PGS than FISH as it can analyze all 24 chromosomes in a single cell with only 1.9% error rate [44, 46]. Using this method, Ata and colleagues clearly demonstrated that female age is main factor determining the aneuploidy rate in both cleavage-stage embryos and blastocysts. Interestingly, increased cohorts did not impact embryo euploidy rates, but actually higher number of embryos (≥7) were associated with the presence of a minimum of one euploid embryo available for transfer in the vast majority of patients across all age groups [23].

The ability of oocyte to be fertilized is largely determined by morphological and functional changes that are controlled by hormonal events, specifically fluctuation of LH and FSH, which work synergistically to regulate the follicle development [8]. The biological activity of FSH is decided by the attachment of carbohydrate moieties, forming heterodimers [8]. However, the degree of gonadotropins' glycosylation is differentially regulated depending on the steroidal status, and therefore, the pattern of circulating FSH during the menstrual cycle is related both to its quantity (concentration) and isoform distribution (quality) [57]. By receptor mediated binding the G protein-coupled receptors present on the granulosa cells (GCs), the gonadotropins containing FSH stimulate the recruitment and growth of early antral follicles (2–5 mm in diameter) [8]. In response to this, activation of adenylate cyclase-mediated signal ensues. Following this, the multiple mRNAs are expressed that encode proteins responsible for cell proliferation, differentiation, and function [58]. The relative proportions of isoforms in human FSH products, either urinary-derived hFSH (u-hFSH) or r-hFSH, depend on the manufacturing process and the origin of the raw material. More acidic isoforms are isolated from urinary products that contain higher numbers of sialic acid residues and have a longer half-life than the more basic isoforms. The more basic isoforms are recombinant molecules that demonstrate higher receptor binding affinity than the more acidic isoforms [59]. On the contrary, increasing the FSH levels in the cells during the first days of stimulation, downregulation of FSH receptors in the granulosa cells has been observed (since the follicular diameter increases ~10 mm) [60].

Exogenous gonadotropins are used to stimulate the ovaries to grow follicles producing oocytes. As a result of the specific isoform profile and gonadotropin content, different commercial preparations may deliver different qualitative and/or quantitative signals to the follicle and contribute to different clinical efficacy outcomes [61]. In unselected patients undergoing IVF, an average of three additional oocytes was obtained with recombinant preparations compared with urinary preparations, resulting in more embryos produced with less total gonadotropin dose [24]. In poor responders, fair evidence indicates that addition of r-LH to r-hFSH preparations would further improve results, as approximately one additional oocyte was obtained by using this aforementioned drug regimen [24]. In a decision-making case-control matched study on the use of combination protocols that compared a fixed ratio of 2 : 1 r-hFSH plus r-hLH or hMG, either alone or in combination with r-hFSH, after downregulation in a long GnRH-agonist protocol in 4,719 women, it was observed that supplementation with r-hLH was significantly more effective. The quantity of oocytes harvested was higher in the r-hFSH and r-hLH combination group [62]. In this study, consumption of r-hFSH was lower (*p* < 0.001) with higher pregnancy rates per cycle (25.5% versus 21.5%; *p* = 0.006; for urinary-derived hMG (u-hMG) alone and 21.7% for u-hMG + r-hFSH; *p* = 0.022) and per embryo transfer (31.3% versus 26.0% for u-hMG alone; *p* = 0.025 and 25.6% for u-hMG + r-hFSH; *p* = 0.008) and implantation per embryo transferred (19% versus 13.9% and 13.8% for u-hMG alone and u-hMG + r-hFSH, resp.; both *p* < 0.001) in the group treated with the fixed combination of r-hFSH and r-hLH. As far as pregnancy is concerned, the literature is rich in meta-analyses comparing efficacy of different gonadotropin products (reviewed by Leão and Esteves) [8]. Despite the heterogeneity pertaining the different stimulation strategies and fertilization method, the overall conclusion is that both urinary gonadotropins, mainly hMG preparations, and r-FSH have similar efficacy in terms of achieving a pregnancy or live birth per treatment cycle. Collectively, these findings translate two principles on stimulation, namely, the issue that r-hFSH is more potent on its receptor than u-FSH and the fact that LH activity (LH or hCG driven) seems to have a role that affects pregnancy outcome.

Premature P rise in non-GnRH analogue cycles has been related to poor oocyte maturation, decreased fertilization, and impaired embryo quality [63]. This phenomenon is related to an early preovulatory LH elevation [64, 65], which also leads to endometrial asynchrony and may affect implantation and OPR. In contrast, serum P levels mainly reflect P output of granulosa cells in GnRH agonist and antagonist cycles as the pituitary is suppressed [14, 66, 67].

In fact, prior to ovulation, granulosa cells in the intrafollicular compartment under LH regulation produce >95% P [68]. In preovulatory follicles, small follicles are “androgenic,” while larger follicles are “estrogenic” and “progestogenic.” The shift from estrogenic to progestogenic state does not involve increase in the concentration of P and this shift is consistent with an increased steroidogenesis by granulosa cells as well as an increased LH sensitivity of granulosa cells [69].

P rise on or before the day of hCG administration occurs in 5–30% of COH with GnRH analogue suppression and has been associated with an increased quantity of retrieved oocytes and the potency of gonadotropin used for COH [27–29, 70]. In case of recombinants, rise in P is likely to be more pronounced, as more mature follicles (more granulosa cells) are recruited, while with urinaries, follicle recruitment is less and follicle recruited is a mixture of mature and atretic follicles. Although some authors have suggested that LH activity driven by hCG content in hMG preparations supports the transformation of intrafollicular P to estradiol, thus increasing endometrium receptivity to embryo implantation, the key enzyme cytochrome 17a-hydroxylase-C17, 20 lyase (P450-17*α*) that converts P to estradiol is virtually absent in the intrafollicular compartment [66, 68, 71]; hence P conversion to estradiol is negligible in humans [72].

Conflicting views have emerged on the impact of P rise measured on the day of hCG administration to implantation in fresh transfers. Some researchers have reported that raised P levels (>1.5 ng/mL) would be detrimental and thus a “freeze-all” embryos policy should be adopted, while others suggested that elevated P levels are associated with a good ovarian response and no detrimental effect on implantation rates [13, 20, 34, 73–75]. The P threshold in which implantation would be affected is also a matter of debate. Some authors indicated that the chances for a successful pregnancy outcome are decreased if P levels are 0.8 ng/mL or greater [75], while others noted that different thresholds should be considered according to the ovarian response [27]. It has also been shown that elevated P levels are not detrimental to implantation in high responders [14, 28]. In such patients, it has been hypothesized that good embryo quality counteracts any detrimental effect of elevated P levels to endometrial receptivity [28]. However, in women with poor ovarian response, both factors act synergistically and therefore, pregnancy rates might be reduced [67].

Along the same lines, recent observations have suggested that other markers would better reflect the impact of a P rise on implantation, including the duration of P elevation, P to estradiol ratio, and P to follicle ratio [18, 76, 77]. In a recent study, Lee and colleagues reported that the clinical pregnancy rate dropped with increase in duration of P elevation (39.6% in women with no P elevation, 38.9% with 1 day of P elevation, 27.3% with 2 days of P elevation, and 0% with 3 days of P elevation) on or before the day of LH surge [77]. In another study, Shufaro and colleagues analyzed 8,649 IVF treatments in normal responders and compared the association of total blood P level and calculated progesterone-to-follicle index (PFI; calculated by dividing blood P to the total number of follicles ≥14 mm) with the clinical pregnancy rate [18]. They observed that PFI was significantly better correlated with IVF cycle outcome than blood P levels (*p* < 0.0001). Elevated P levels led to a lower pregnancy rate only if the P levels were >93 percentile. The authors concluded that an elevation in P levels due to an increased number of follicles is not as important as high PFI, which could negatively affect the pregnancy rate.

In conclusion, there is a lack of clarity among IVF specialists regarding adopting “freeze-all” policy in all cycles with levels of serum P >1.5 ng/mL on day of hCG administration. The P levels measured in blood represent the sum of P secreted by multiple follicles. Given the supraphysiological stimulation of granulosa cells, there may be more P in circulation at the late follicular phase as about 95% of the circulating P is produced by GCs in the ovarian intrafollicular compartment. P levels > 1.5 ng/mL do not necessarily relate to worsening of cycle outcomes, especially in patients with high cohorts. COH regimens with LH activity are not useful to reduce serum P because its conversion to estradiol is negligible owing to a lack of the enzyme that drives this pathway.

The recommendations of the expert panel on all these issues are listed in Figure 1.

## 5. Conclusions

Clinical pregnancy rates are still considered as the primary goal and surrogate marker of IVF success by many practitioners [2, 3], despite firm evidence indicating that the most important goal of ART is to attain healthy offspring [78, 79]. Therefore, hormonal stimulation of the ovaries should be refined to improve effectiveness and safety, allowing the creation of longitudinal families by elective transfer of reduced number of embryos [40]. Considering the importance of the number of oocytes on LBR, cumulative pregnancy rate is probably the most relevant pregnancy endpoint in ART.

The optimum number of oocytes that delivers the highest LBR per treatment is 10–15. Maternal age but not cohort size affects embryo quality. An increased embryo cohort is associated with the higher availability of euploid embryos for transfer in all age groups. Whenever possible, COS should be designed to achieve these goals. More acidic isoforms are isolated from urinary products that contain higher numbers of sialic acid residues and have a longer half-life than the more basic isoforms. The more basic isoforms are recombinant molecules that demonstrate higher receptor binding affinity than the more acidic isoforms. Different commercial preparations may deliver different qualitative and/or quantitative signals to the follicle and contribute to different clinical efficacy outcomes due to isoform profile and gonadotropin content. Conflicting evidence exists on the effect of P elevation determined on the day of hCG administration to implantation in fresh transfer cycles. P elevation does not necessarily impair cycle outcomes, especially in patients with high cohorts. Therefore, a “freeze-all” policy should not be adopted in all cycles with serum P of 1.5 ng/mL or greater on the day of hCG. However, further research is still needed in this area.

## Figures and Tables

**Table 1:** Characteristics of included studies addressing the questions under investigation.

Authors, year	Methods	Patient population	Interventions/methods	Question under study
Sunkara et al., 2011	Analysis of the United Kingdom IVF Registry database from April 1991 to June 2008	400,135 IVF cycles with autologous oocytes and fresh transfers	To explore the association between the number of eggs and live birth outcome, a likelihood logistic model with live birth outcome as the dependent variable was fitted using a fractional polynomial to handle the number of oocytes as a continuous independent variable	1

Ji et al., 2013	Cohort study	Normogonadotropic women (*n* = 2,455) aged 18–34 years undergoing their first IVF treatment cycle with a long GnRH agonist protocol at a single institution, including one complete treatment round of a fresh transfer cycle and subsequent frozen transfer cycles	Patients were categorized into four groups according to the number of oocytes retrieved: 0–5, 6–10, 10–15, or ≥16 oocytes. LBR per fresh transfer and cumulative LBR were calculated per group and compared. Logistic regression analyses identified association between oocyte number and live birth outcome after adjusting for confounders	1

De Geyter et al., 2015	Analysis of the Swiss IVF Registry database from 1993 and 2012	>100,000 IVF cycles reporting delivery rates per fresh embryo transfer	Data extraction and analysis of IVF outcomes, including graphical displays with the crude outcome data	1

Ata et al., 2012	Cohort study	990 patients undergoing IVF in 70 North American Centers between January 2010 and July 2011	PGS of human embryos by array CGH. Embryo biopsy was performed on day 3 or at blastocyst stage. The proportion of euploid embryos over embryos biopsied was calculated per cycle. Linear regression analysis was performed to assess the effect of cohort size on euploidy rate adjusted for the effect of female age	2

Lehert et al., 2010	Systematic review and meta-analysis of prospective randomized or quasi-RCT	16 studies involving 4,040 women aged 40 or less undergoing IVF or ICSI irrespective of use of GnRH agonists or antagonists in which hMG and r-hFSH for COS were compared	Comparison of number of retrieved oocytes (primary endpoint) between two modalities of gonadotropin treatment, using the random effects model. Evidence of superiority of one treatment was accepted when the results of the main analysis and the sensitivity analyses were consistent	3

Lehert et al., 2014	Systematic review and meta-analysis of prospective, randomized, parallel-, comparative-group trials	40 RCTs involving 6,443 patients aged 18-45 undergoing IVF or ICSI treated with GnRH analogues and r-hFSH plus r-hLH or r-hFSH alone for COS	Comparison of number of retrieved oocytes and CPR between two modalities of gonadotropin treatment (primary endpoints), using the random effects model. Ovarian response to treatment—normal or poor—was included as a covariate for subgroup analysis	3

Xu et al., 2012	Retrospective cohort study	11,055 consecutive patients undergoing IVF/ICSI using long GnRH agonist protocol and a subgroup of 4,021 patients participating in a FET program at a single institution from January 2002 and September 2011	OPRs assessed with logistic regression analysis according to 8 distinct P levels intervals on the day of hCG administration and compared among low (<4 oocytes retrieved), intermediate (5–19 oocytes retrieved), and high (≥20 oocytes retrieved) responders. P thresholds for a detrimental effect on cycle outcome were calculated	4

Griesinger et al., 2013	Retrospective combined analysis from six clinical trials comparing GnRH agonists and antagonists in COS	Normogonadotropic women (*n* = 1,866) up to 39 years of age undergoing COS with r-hFSH and GnRH antagonist	OPRs assessed with univariate and multivariate analyses according to serum P levels ≤1.5 ng/mL versus >1.5 ng/mL on the day of hCG administration and compared among low (1–5 oocytes retrieved), normal (6–18 oocytes retrieved), and high (>18 oocytes retrieved) responders	4

Requena et al., 2014	Retrospective cohort study	2,850 high responders (≥20 oocytes retrieved or estradiol levels ≥3000 pg/mL) undergoing IVF-ET or FET in 11 Spanish institutions from January 2009 to December 2011	Implantation and CPRs assessed with logistic regression analysis according to 5 distinct serum P levels intervals on the day of hCG administration	4

Venetis et al., 2015	Retrospective cohort study	3,296 patients undergoing IVF/ICSI and fresh ET in a single IVF center between 2001 and 2013	LBRs assessed with bivariate and multivariate analyses according to serum P levels ≤1.5 ng/mL versus >1.5 ng/mL on the day of hCG administration and compared among low (<6 oocytes), normal (6–18 oocytes), and high (>18 oocytes) responders	4

Questions under study are as follows. (1) What is the optimum number of oocytes that is associated with the highest live birth rates? (2) Is there a correlation between cohort size and embryo quality? (3) Does the choice of gonadotropins affect oocyte yield in IVF? (4) Should a “freeze-all” policy be adopted in all cycles with progesterone levels >1.5 ng/ml on day of hCG?

IVF: in vitro fertilization; LBR: live birth rates; PGS: preimplantation genetic screening; CGH: comparative genomic hybridization; RCT: randomized controlled trials; ICSI: intracytoplasmic sperm injection; hMG: human menopausal gonadotropin; r-hFSH: recombinant human follicle stimulating hormone; COS: controlled ovarian stimulation; r-hLH: recombinant human luteinizing hormone; CPR: clinical pregnancy rates; FET: frozen-embryo transfer; GnRH: gonadotropin releasing hormone; P: progesterone; OPR: ongoing pregnancy rate; ET: embryo transfer; hCG: human chorionic gonadotropin.
